# *Clonostachys* *allendensis* sp. nov.: A New Species Isolated from Bean Straw in Durango, Mexico

**DOI:** 10.3390/microorganisms14091962

**Published:** 2026-09-05

**Authors:** Raúl Alejandro Cuevas-Jacquez, Esperanza Herrera-Torres, Rocio Aide Carrasco-Rubio, Olga Miriam Rutiaga-Quiñones, Elia Esther Araiza-Rosales, Lily Xochilt Zelaya-Molina

**Affiliations:** 1 Institutional Doctorate in Agricultural and Forestry Sciences (DICAF), Universidad Juárez del Estado de Durango (UJED), Durango 34113, Mexico; racj.78@gmail.com (R.A.C.-J.); rocioa.carrub@gmail.com (R.A.C.-R.); 2 Tecnológico Nacional de México, Instituto Tecnológico del Valle del Guadiana (TecNM-ITVG), Durango 34371, Mexico; hetoes99@yahoo.com.mx; 3 Tecnológico Nacional de México/Instituto Tecnológico de Durango, Departamento de Ingenierías Química y Bioquímica, Laboratorio Nacional CONAHCYT para la Evaluación de Productos Bióticos, LANAEPBi, Felipe Pescador 1830 Ote, Colonia Nueva Vizcaya, Durango 34180, Mexico; omrutiaga@itdurango.edu.mx; 4 Secretaría de Ciencia, Humanidades, Tecnología e Innovación-Facultad de Medicina Veterinaria y Zootecnia, Universidad Juárez del Estado de Durango, Durango 34307, Mexico; 5 Centro-Nacional de Recursos Genéticos-INIFAP, Tepatitlan 47600, Mexico

**Keywords:** Bionectriaceae, multilocus phylogeny, taxonomy, morphology

## Abstract

The genus *Clonostachys* comprises morphologically similar species whose delimitation often requires integrative taxonomic approaches. In this study, three fungal strains (RC2, RC5, and RC7) isolated from pinto bean (*Phaseolus vulgaris* L.) straw collected in the Los Llanos region of Durango, Mexico, were examined using morphological and multilocus phylogenetic analyses. Phylogenetic reconstruction based on five loci (ITS, LSU, *TEF1*, *TUB2*, and *RPB2*) consistently resolved the isolates as a single, strongly supported monophyletic lineage distinct from closely related species, including *Clonostachys rhizophaga*, *C. chloroleuca*, and *C. kunmingensis*. Morphologically, *Clonostachys allendensis* is distinguished from its closest phylogenetic relatives by a combination of yellowish perithecia, cylindrical to slightly ampulliform phialides, predominantly unicellular ellipsoidal conidia, and pale pink to salmon colony pigmentation. In addition, multilocus phylogenetic analyses consistently recovered *C. allendensis* as a distinct and well-supported lineage within *Clonostachys*. Among the isolates examined, strain RC7 (CM-CNRGᵀ) was designated as the type strain. Based on the combined molecular and morphological evidence, this lineage is herein described as *Clonostachys allendensis* sp. nov. This study expands current knowledge of *Clonostachys* diversity in Mexico and contributes to the understanding of fungal diversity associated with agricultural residues.

## 1. Introduction

The genus *Clonostachys* Corda (Bionectriaceae, Hypocreales) comprises a diverse group of fungi with a cosmopolitan distribution, with greater diversity reported from tropical and subtropical regions [1]. Historically, *Clonostachys* has been associated with the sexual genus *Bionectria*, and both taxa were treated as conspecific in several taxonomic studies [2,3]. Following the adoption of the “One Fungus–One Name” principle, the name *Clonostachys* was retained because of its nomenclatural priority and widespread use, thereby promoting taxonomic stability within the group [4]. Species of *Clonostachys* exhibit broad ecological versatility and have been reported as saprotrophs, endophytes, mycoparasites, entomopathogens, and phytopathogens in diverse natural and agricultural ecosystems [1,5,6,7]. In addition to their ecological importance, several species have attracted increasing attention because of their enzymatic potential and possible applications in biological control and lignocellulosic biomass degradation. These diverse ecological and functional attributes highlight the importance of accurately characterizing and delimiting species within the genus.

Although species of *Clonostachys* can be differentiated based on features of the sexual and asexual morphs, including ascomata, asci, conidiophores, and conidia, species delimitation is often complicated by phenotypic plasticity and morphological overlap among closely related taxa [2,4]. Consequently, integrative taxonomic approaches combining morphology with multilocus phylogenetic analyses have become essential for accurate species recognition and for revealing cryptic diversity within the genus [1]. Multilocus phylogenetic approaches are particularly valuable for delimiting closely related fungal species because individual molecular markers may differ in their evolutionary rates and phylogenetic resolution. Individual loci may also differ in their phylogenetic signal and ability to resolve relationships among recently diverged lineages. The nuclear ribosomal internal transcribed spacer (ITS) region is recognized as the primary DNA barcode for fungi; however, additional ribosomal and protein-coding loci can provide complementary phylogenetic and discriminatory information for resolving relationships among closely related taxa [8]. Within *Clonostachys*, multilocus approaches combining ITS and LSU with protein-coding markers such as *TEF1*, *TUB2*, and *RPB2* provide a more robust framework for assessing phylogenetic relationships and species boundaries among closely related taxa [1].

The examination of individual gene genealogies provides an additional framework for assessing whether putative species represent independently evolving lineages. Genealogical concordance phylogenetic species recognition (GCPSR) evaluates concordance among multiple independent gene genealogies and provides an operational approach for recognizing phylogenetic species in fungi [9]. Under this framework, concordance among independent loci can strengthen evidence for lineage independence, whereas strongly supported conflicts among gene trees require careful evaluation [9]. Because individual loci may differ in their phylogenetic signal and resolution, species delimitation should not rely exclusively on a single marker or require identical topologies among all gene trees. Instead, genealogical evidence can be evaluated in combination with multilocus phylogenetic inference, sequence-level divergence, and diagnostic morphological characters to provide complementary evidence for species recognition [1,9].

In Mexico, studies on *Clonostachys* diversity remain limited, and most reports have focused primarily on species associated with soil, plant residues, or biological control applications. Consequently, the taxonomic diversity and ecological distribution of the genus across different agricultural regions of the country remain poorly documented. During a survey of fungi associated with pinto bean (*Phaseolus vulgaris* L.) straw collected in the Los Llanos region of Durango, Mexico, isolates morphologically consistent with *Clonostachys* were recovered and subjected to morphological and multilocus phylogenetic analyses. The aim of this study was to determine the taxonomic identity and phylogenetic placement of these isolates through an integrative approach combining macro- and micromorphological characterization with multilocus phylogenetic analyses based on ITS, LSU, *TEF1*, *TUB2*, and *RPB2*. Genealogical concordance among individual loci and sequence-level divergence from phylogenetically related species were additionally evaluated to provide complementary evidence for species delimitation. The combined morphological and molecular evidence supports the recognition of these isolates as a distinct species, which is herein described as *Clonostachys allendensis* sp. nov. This study expands current knowledge of *Clonostachys* diversity in Mexico and contributes to the understanding of fungal diversity associated with agricultural residues.

## 2. Materials and Methods

### 2.1. Sampling, Isolation, and Morphological Characterization

During the autumn–winter season of 2023, pinto bean (*Phaseolus vulgaris* L., cv. Pinto Saltillo) straw was collected from the Santa Catalina de Siena site, located in the municipality of Guadalupe Victoria, Durango, Mexico (24.58° N, 104.09° W). The straw was ground to an 8 mm particle size, and 30 g of material was placed into 100 mL Erlenmeyer flasks. Moisture content was adjusted to 65%, and the flasks were incubated under dark conditions at room temperature for 10 days to promote fungal development [10].

Fragments of colonized straw were aseptically transferred onto potato dextrose agar (PDA) supplemented with ampicillin and incubated at 28 °C for 72 h. A total of six fungal isolates were recovered and purified through successive subcultures on PDA until axenic cultures were obtained [11]. Based on preliminary morphological observations, three isolates (RC2, RC5, and RC7) exhibiting characteristics consistent with *Clonostachys* were selected for detailed taxonomic analyses.

Macromorphological characterization included colony color, texture, margin, growth pattern, and surface appearance. Micromorphological observations focused on reproductive and vegetative structures, including conidiophores, phialides, conidia, perithecia, asci, and ascospores when present. Observations were performed using a LEICA DM750^®^ microscope (Wetzlar, Germany) equipped with a LEICA ICC50HD^®^ camera. Colony colors were described according to the Royal Botanic Garden Edinburgh Fungal Colour Chart [12].

### 2.2. Molecular Identification

Genomic DNA extraction, PCR amplification, purification, and sequencing were performed by Macrogen Genomic Services (Seoul, Republic of Korea). Five loci commonly used in fungal systematics were amplified and sequenced: the internal transcribed spacer region (ITS), the large subunit ribosomal RNA gene (LSU), translation elongation factor 1-alpha (*TEF1*), beta-tubulin (*TUB2*), and the second-largest subunit of RNA polymerase II (*RPB2*). These loci were selected to provide complementary phylogenetic information from ribosomal and protein-coding regions and to improve species-level resolution within *Clonostachys* [1]. Forward and reverse chromatograms were inspected and edited using FinchTV v. 1.4.0, and consensus sequences were assembled in BioEdit v. 7.2.5. Preliminary taxonomic identification was performed through BLASTn v. 2.17.0 searches against sequences available in the GenBank database. The resulting consensus sequences were subsequently incorporated into the multilocus phylogenetic dataset together with reference sequences representing phylogenetically related species of *Clonostachys* and selected outgroup taxa.

### 2.3. Sequence Alignment and Phylogenetic Analyses

Sequences generated in this study, together with reference sequences retrieved from GenBank^®^, were aligned and inspected in BioEdit and MEGA7 [13]. Reference taxa were selected prim arily according to recent comprehensive phylogenetic treatments of *Clonostachys* and related genera [1]. Each locus (ITS, LSU, *RPB2*, *TEF1*, and *TUB2*) was aligned separately prior to concatenation, and the resulting alignments were inspected to verify sequence homology and remove ambiguous terminal regions when necessary. The five individual alignments were subsequently concatenated in the order ITS, LSU, *RPB2*, *TEF1*, and *TUB2*. The final concatenated dataset comprised 70 taxa and 4029 aligned nucleotide positions. GenBank accession numbers for all strains and loci included in the analyses are provided in Table 1. Maximum Likelihood (ML) phylogenetic inference of the concatenated dataset was performed using IQ-TREE v3.1.3 [14]. The dataset was analyzed under a partitioned framework in which ITS, LSU, *RPB2*, *TEF1*, and *TUB2* were treated as independent partitions. ModelFinder [15], implemented in IQ-TREE, was used to determine the best-fitting nucleotide substitution model for each partition according to the Bayesian Information Criterion (BIC). Branch support was evaluated using 1000 ultrafast bootstrap replicates [16]. The resulting ML tree was rooted using selected species of *Sesquicillium* as outgroup taxa.

Additionally, to evaluate genealogical concordance among loci, separate ML phylogenetic analyses were also conducted for ITS, LSU, *RPB2*, *TEF1*, and *TUB2* in MEGA7; the evolutionary model was selected according to BIC, and the branch support was estimated with 500 bootstrap replicates. The resulting single-locus topologies were compared under the genealogical concordance phylogenetic species recognition (GCPSR) framework [9], with particular attention to the placement of RC7 relative to RC2 and RC5 and to the closest phylogenetic relatives, including *C. rhizophaga*, *C. kunmingensis*, and *C. chloroleuca*. Genealogical discordance was evaluated considering both topological differences and the bootstrap support associated with conflicting relationships. For the GCPSR assessment, a genealogy was considered concordant when RC2, RC5, and RC7 were recovered as an exclusive lineage, whereas conflicting relationships were evaluated according to both their topology and associated bootstrap support. Genealogies with low nodal support were treated as unresolved rather than as strong evidence of genealogical conflict.

A complementary Bayesian inference was performed in MrBayes v3.2.7a [17] using the same five-locus concatenated dataset and partition scheme; substitution models were specified independently for each partition as SYM + I + G for ITS, GTR + I + G for LSU, *RPB2*, and *TEF1*, and HKY + I + G for *TUB2*, with model parameters unlinked across partitions. Two independent Markov chain Monte Carlo (MCMC) runs, each consisting of four chains, were conducted for 15,000,000 generations, with trees and model parameters sampled every 1000 generations. The first 25% of samples were discarded as burn-in. Convergence between independent runs was evaluated using effective sample sizes (ESSs), potential scale reduction factors (PSRFs), and split-frequency diagnostics. Posterior probabilities (PPs) were calculated from the post-burn-in trees and used as complementary measures of nodal support. Finally, molecular divergence between RC7 and selected phylogenetically related species (*C. rhizophaga*, *C. kunmingensis*, and *C. chloroleuca*) was evaluated independently for each locus in MEGA7 [13]. Absolute nucleotide differences were calculated using the “No. of differences” option, whereas uncorrected p-distances were estimated using pairwise deletion of gaps and missing data. These analyses were used as quantitative complements to the phylogenetic and morphological evidence for species delimitation. All DNA sequences obtained in this study were deposited in GenBank.

## 3. Results

### 3.1. Multilocus Phylogeny

**Figure 1:**
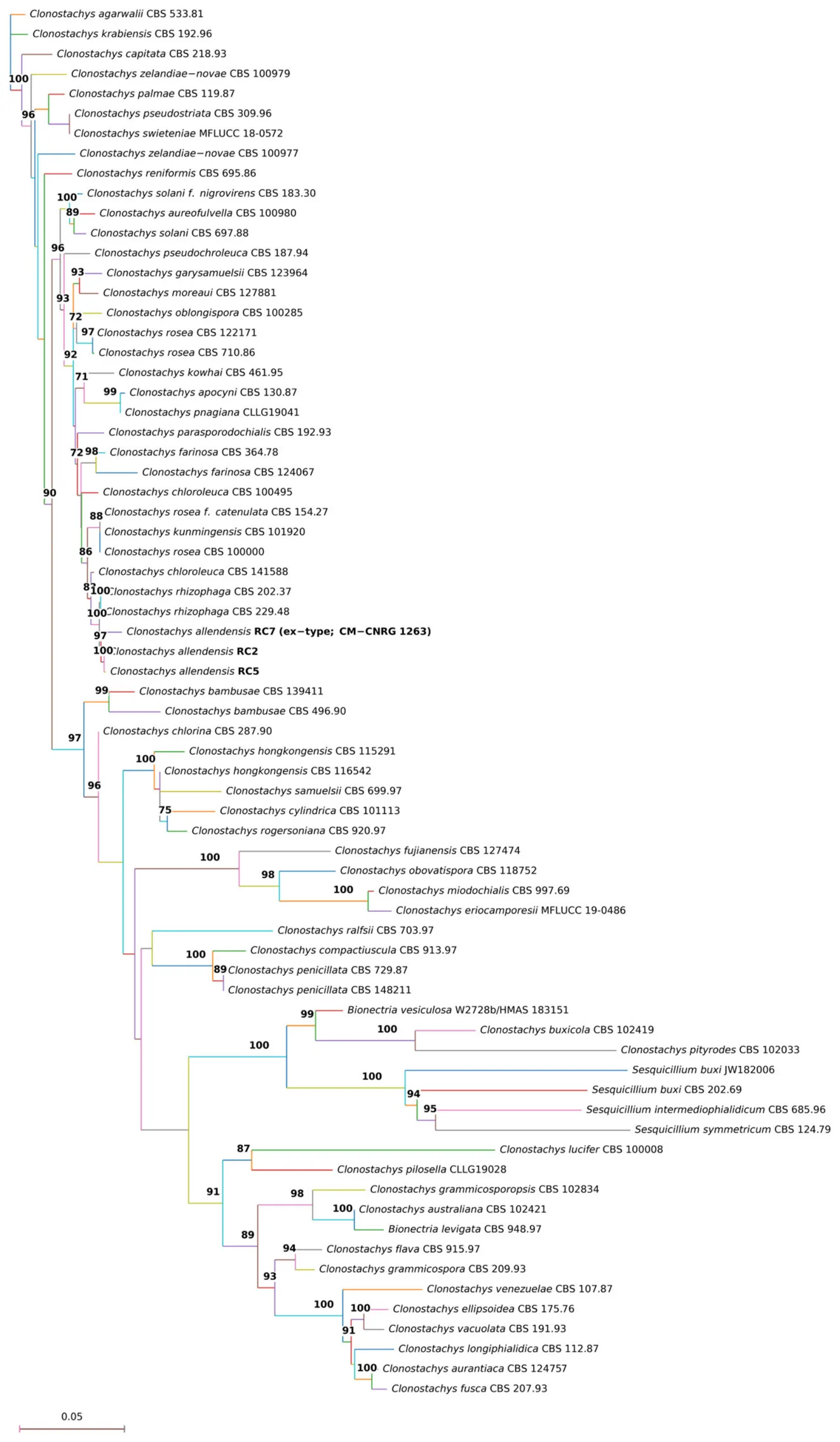
Maximum Likelihood (ML) phylogenetic tree inferred from the concatenated ITS, LSU, *RPB2*, *TEF1*, and *TUB2* dataset comprising 70 taxa and 4029 aligned nucleotide positions. Phylogenetic reconstruction was performed in IQ-TREE using a partitioned analysis, with the best-fitting substitution model for each locus selected by ModelFinder according to the Bayesian Information Criterion (BIC). Branch support was assessed using 1000 ultrafast bootstrap replicates (UFBoot); only support values ≥ 70% are shown at the nodes. *Sesquicillium* species were included as outgroup taxa. Branch lengths are proportional to the number of nucleotide substitutions per site, as indicated by the scale bar.

**Figure 2:**
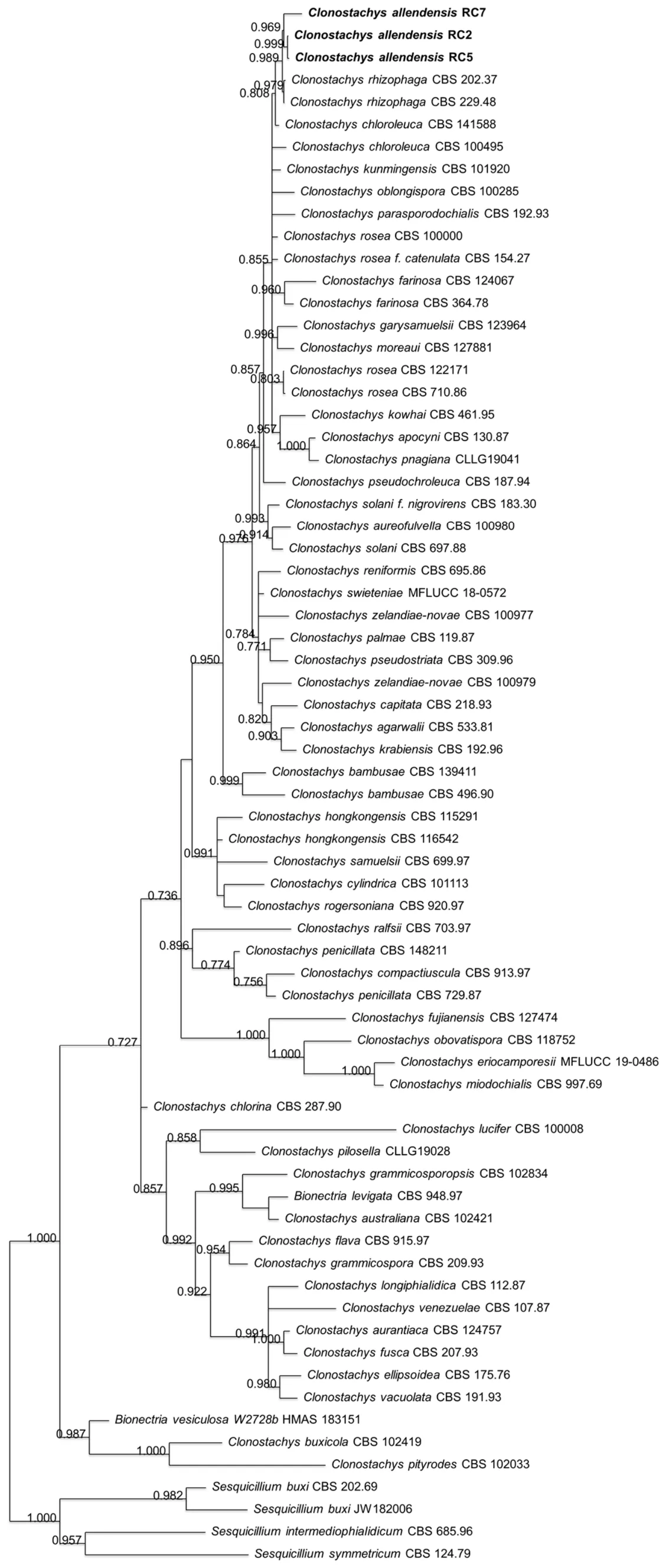
Bayesian majority-rule consensus phylogeny inferred from the concatenated ITS, LSU, *RPB2*, *TEF1*, and *TUB2* dataset using a partitioned analysis in MrBayes. Substitution models applied to the individual partitions were SYM + I + G for ITS, GTR + I + G for LSU, *RPB2*, and *TEF1*, and HKY + I + G for *TUB2*. Nodal labels indicate posterior probabilities (PPs); branch lengths are proportional to the expected number of nucleotide substitutions per site, as indicated by the scale bar. *Sesquicillium* spp. were used as the outgroup.

The final concatenated dataset based on ITS, LSU, *RPB2*, *TEF1*, and *TUB2* sequences comprised 70 taxa representing *Clonostachys* species and related taxa, including the three isolates obtained from pinto bean straw in Durango, Mexico. The concatenated alignment contained 4029 nucleotide positions. The partitioned Maximum Likelihood (ML) analysis performed in IQ-TREE recovered RC2, RC5, and RC7 as a distinct monophyletic lineage with 97% ultrafast bootstrap (UFBoot) support (Figure 1). Within this lineage, RC2 and RC5 formed a strongly supported internal clade (100% UFBoot), whereas RC7 occupied a distinct position within the same lineage. The RC2–RC5–RC7 lineage was phylogenetically differentiated from closely related species, including *C. rhizophaga*, *C. kunmingensis*, and *C. chloroleuca*. Bayesian inference independently recovered the same RC2–RC5–RC7 lineage with a posterior probability of PP = 0.969, while the RC2 + RC5 subclade received PP = 0.999. The two reference strains of *C. rhizophaga* formed a separate clade (PP = 0.979), and the node connecting the *C. rhizophaga* clade with the RC2–RC5–RC7 lineage received PP = 0.989 (Figure 2). The congruent recovery of the proposed lineage under Maximum Likelihood and Bayesian inference provided complementary support for its phylogenetic coherence. Single-locus Maximum Likelihood analyses of ITS, LSU, *RPB2*, *TEF1*, and *TUB2* were additionally examined under the genealogical concordance phylogenetic species recognition (GCPSR) framework (Supplementary Figures S1–S5). ITS, LSU, *RPB2*, and *TEF1* recovered RC2, RC5, and RC7 as an exclusive lineage, although bootstrap support varied among loci. Among the individual loci, *TEF1* provided the strongest support for the lineage (86.8% bootstrap support).

In contrast, the *TUB2* genealogy showed topological discordance because the broader clade containing RC2, RC5, and RC7 also included *C. rhizophaga.* However, the immediate relationship recovered between RC7 and *C. rhizophaga* CBS 202.37 received only 4.75% bootstrap support. Therefore, the single-locus analyses indicated predominant genealogical concordance, with limited topological discordance in *TUB2*, but without a strongly supported conflicting sister relationship involving RC7. The genealogical relationships recovered for RC7 across the five individual loci are summarized in Table 2.

Sequence-level comparisons provided an additional quantitative assessment of the molecular differentiation of RC7 from its closest phylogenetic relatives (Table 3). Depending on the reference strain available for each locus, RC7 differed from *C. rhizophaga* by 2–4 nucleotide substitutions in ITS, 4 in LSU, 2 in *RPB2*, 2 in TEF1, and 9 in *TUB2*. Relative to *C. kunmingensis*, RC7 differed by 4 substitutions in ITS, 5 in LSU, 17 in *RPB2*, 5 in *TEF1*, and 16 in *TUB2*. Comparisons with *C. chloroleuca* revealed 4 substitutions in ITS, 7 in LSU, 6 in *RPB2*, 8 in *TEF1*, and 30 in *TUB2*. Corresponding uncorrected p-distances are presented in Table 3.

Taken together, the partitioned Maximum Likelihood analysis, Bayesian inference, individual-locus genealogies, and sequence-level comparisons provided complementary evidence for the phylogenetic distinctiveness of the lineage represented by RC2, RC5, and RC7. Although complete genealogical concordance was not recovered across all five loci, four loci supported an exclusive RC2–RC5–RC7 lineage and the only topologically discordant locus, *TUB2*, did not recover a strongly supported conflicting sister relationship involving RC7. These molecular results, considered together with the morphological differences described below, support the recognition of this lineage as *Clonostachys allendensis* Cuevas-Jacquez et al., sp. nov.

### 3.2. Taxonomy of Clonostachys allendensis sp. nov.

Mycobank number: MB863532.

Etymology: The specific epithet *allendensis* refers to Ignacio Allende, a locality within the municipality of Guadalupe Victoria, Durango, Mexico. The name recognizes the geographic region where the species was discovered, including the neighboring locality of Santa Catalina de Siena, from which the type strain was isolated.

Typification: Holotype: MEXICO, Durango, municipality of Guadalupe Victoria, Santa Catalina de Siena; isolated from pinto bean straw (*Phaseolus vulgaris* L.); autumn–winter season 2023; coll. R.A. Cuevas-Jacquez; cryopreserved culture RC7, permanently preserved in a metabolically inactive state in the public Culture Collection of the National Center for Genetic Resources (CM-CNRG, INIFAP), accession CM-CNRG 1263 [18]. Ex-holotype living culture: RC7. GenBank accession numbers: ITS, PQ596151; LSU, PX400600; *TEF1*, PX436934; *TUB2*, PX436937; *RPB2*, PX436940.

**Figure 3:**
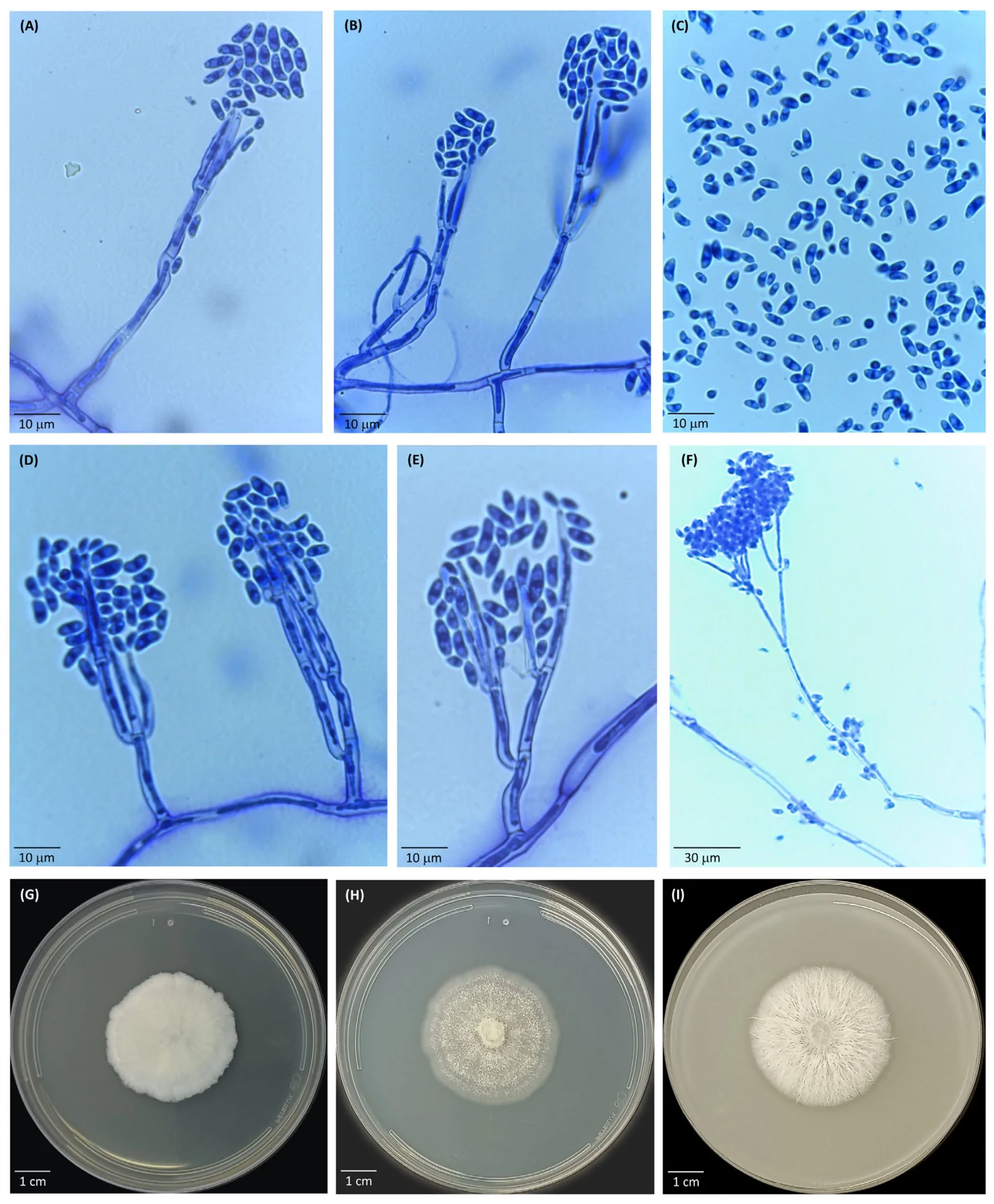
Macro- and micromorphology of the holotype *Clonostachys allendensis*. (**A**–**F**) Micro-morphological features of the holotype *C. allendensis*. (**A**,**B**) Erect, septate conidiophores with terminal verticils of phialid. (**C**) Abundant, smooth, hyaline, ellipsoidal conidia. (**D**,**E**) Subulate phialide forming dense heads and producing acropetal chains of conidia. (**F**) Branched conidiophores bearing multiple verticils and clusters of freshly formed conidia. (**G**–**I**) Colony morphology on different media: (**G**) PDA, dense and cottony colony; (**H**) SNA, thin mycelium with a compact center; (**I**) OA, aerial mycelium with a radial pattern. *Clonostachys chloroleuca* is characterized by consistently greenish conidial masses on artificial media, contrasting with the pale pink to salmon colony pigmentation observed in *C. allendensis* [19].

Colony morphology: On PDA, colonies were slow-growing, with dense cottony to floccose aerial mycelium. Colonies were initially white, later developing pale pink to salmon pigmentation during maturation. The colony surface was homogeneous to slightly zonate, with regular to slightly diffuse margins that occasionally became lobed in older cultures. The reverse side ranged from pale cream to pinkish. On SNA, colonies were thinner and more translucent, with sparse aerial mycelium and reduced pigmentation. On OA, colonies were moderately developed, with more abundant aerial hyphae, radial growth patterns, and occasional concentric zonation. Colony pigmentation and growth rate varied slightly among isolates and according to culture conditions (Figure 3).

Micromorphology: Hyphae hyaline, septate, smooth-walled, branched, 1–4 µm wide. Conidiophores arising laterally or terminally from vegetative hyphae, short to moderately elongated, erect, smooth-walled, simple or irregularly branched. Phialides solitary or arranged in whorls, cylindrical to lageniform or slightly ampulliform, measuring (8–)10–30(–35) µm × 2–4(–5) µm, with inconspicuous collarettes. Conidia unicellular, hyaline, smooth-walled, ellipsoidal to ovoid or subglobose, occasionally ellipsoidal, measuring (2.5–)3–8(–9) µm × (1.5–)2–3(–4) µm, produced abundantly in slimy heads or short chains. Chlamydospores were absent or only rarely observed (Figure 3).

Sexual morph: Sexual structures were observed in some isolates. Perithecia superficial to semi-immersed, solitary or gregarious, globose to ellipsoidal, measuring (120–)150–250(–300) µm in diameter, yellowish to orange-yellow at maturity, smooth to finely roughened. Asci cylindrical to clavate, unitunicate, 8-spored. Ascospores bicellular, hyaline, smooth-walled, ellipsoidal to slightly fusiform, measuring (6–)7–12(–14) µm × (2.5–)3–5(–6) µm, slightly constricted at the septum. Sexual structures developed variably depending on substrate and culture conditions (Figure 3).

Habitat: On pinto bean straw (*Phaseolus vulgaris* L.) collected in the Llanos region of Durango, northern Mexico.

Notes: *Clonostachys allendensis* is distinguished from its closest phylogenetic relatives by a unique combination of morphological and phylogenetic characters (Table 4). The new species develops pale pink to salmon colonies on PDA, whereas *C. kunmingensis* develops pale yellow colonies with a pale orange reverse [1]. In addition, *C. allendensis* produces predominantly unicellular ellipsoidal to ovoid conidia measuring (2.5–)3–8(–9) × (1.5–)2–3(–4) µm, while *C. kunmingensis* produces oblong to cylindrical primary conidia measuring (6–)6.7–11.2(–14) × (2–)2.3–4.5(–4.8) µm and slightly curved secondary conidia measuring (4–)4.2–8.5(–9) × (2.2–)2.5–4(–4.5) µm [1]. Furthermore, *C. allendensis* develops yellowish to orange-yellow perithecia, whereas the sexual morph of *C. kunmingensis* remains unknown [1]. Compared with *C. rhizophaga*, the new species differs in phialide morphology and dimensions, with phialides measuring (8–)10–30(–35) × 2–4(–5) µm, whereas those reported for *C. rhizophaga* measure (15.6–)22.0–34.2(–48.2) µm [4].

Sequence-level comparisons further differentiated RC7 from *C. chloroleuca* across all five loci examined, with 4 nucleotide differences in ITS, 7 in LSU, 6 in *RPB2*, 8 in *TEF1*, and 30 in TUB2 (Table 3). Similarly, RC7 differed from *C. kunmingensis* and *C. rhizophaga* at multiple nucleotide positions across the five loci. These differences were accompanied by locus-specific uncorrected p-distance values (Table 3), providing a quantitative complement to the phylogenetic and morphological evidence.

The molecular differentiation of *C. allendensis* is therefore not based exclusively on the topology of the concatenated phylogeny. The lineage was independently recovered with strong support in the partitioned Maximum Likelihood analysis (97% UFBoot) and Bayesian inference (PP = 0.969), while four of the five individual gene genealogies recovered RC2, RC5, and RC7 as an exclusive lineage. Although *TUB2* showed topological discordance, the conflicting immediate relationship involving RC7 and *C. rhizophaga* CBS 202.37 received only 4.75% bootstrap support. Consequently, the combined evidence from partitioned multilocus phylogenetic inference, Bayesian analysis, predominant genealogical concordance, sequence-level differentiation, and diagnostic morphological characters supports the recognition of *C. allendensis* as a distinct species within *Clonostachys*.

## 4. Discussion

The integration of multilocus phylogenetic analyses and detailed morphological observations provides strong support for the recognition of *Clonostachys allendensis* Cuevas-Jacquez et al., sp. nov. as a distinct species within Bionectriaceae. The isolates examined formed a well-supported monophyletic lineage clearly separated from closely related taxa, including *C. rhizophaga*, *C. chloroleuca*, and *C. kunmingensis*. The concordance between phylogenetic placement and diagnostic morphological traits supports the taxonomic independence of the species and highlights the usefulness of multilocus approaches for resolving species boundaries within *Clonostachys*, a genus known for considerable phenotypic overlap and cryptic diversity [2,4]. In particular, loci such as *TEF1* and *RPB2* have been useful for resolving relationships among closely related taxa within Hypocreales and Bionectriaceaeae [4,20].

The phylogenetic evidence obtained in the present study was further strengthened by the use of complementary analytical approaches rather than relying exclusively on the topology of a single concatenated tree. The partitioned Maximum Likelihood analysis performed in IQ-TREE recovered the RC2–RC5–RC7 lineage with 97% ultrafast bootstrap support, whereas Bayesian inference independently recovered the same lineage with a posterior probability of 0.969. Within this lineage, RC2 and RC5 formed a particularly strongly supported internal clade (100% UFBoot; PP = 0.999), whereas RC7 maintained a distinct position within the same lineage. The congruent recovery of the proposed species under Maximum Likelihood and Bayesian frameworks therefore provides complementary evidence for the stability of its phylogenetic placement.

Evaluation of the five individual gene genealogies provided an additional level of evidence for species delimitation. ITS, LSU, *RPB2*, and *TEF1* recovered RC2, RC5, and RC7 as an exclusive lineage, although bootstrap support varied substantially among loci. Among these markers, *TEF1* provided the strongest individual-locus support (86.8%). In contrast, the *TUB2* genealogy showed topological discordance because the broader clade containing RC2, RC5, and RC7 also included *C. rhizophaga*. However, the immediate relationship recovered between RC7 and *C. rhizophaga* CBS 202.37 received only 4.75% bootstrap support. Thus, this topology does not provide strong evidence for an alternative relationship that would contradict the species hypothesis. Instead, the overall pattern indicates partial genealogical concordance, with no strongly supported single-locus conflict opposing the lineage consistently recovered by the concatenated Maximum Likelihood and Bayesian analyses.

Differences in resolution among individual loci are consistent with the behavior previously reported for multilocus analyses of *Clonostachys*, in which ribosomal and protein-coding markers contribute different levels of phylogenetic information [1,4]. Consequently, the relatively weak support observed in some individual gene trees should not be interpreted in isolation. Rather, species delimitation should consider the predominant signal across independent loci together with the concatenated phylogeny, Bayesian support, sequence-level differentiation, and morphology. The comparatively stronger resolution provided by *TEF1* in the present study is also consistent with its recognized value for resolving relationships among closely related species of *Clonostachys* [1].

Sequence-level comparisons further supported the molecular differentiation of RC7 from phylogenetically related taxa. Depending on the reference strain available for each locus, RC7 differed from *C. rhizophaga* by 2–4 nucleotide positions in ITS, 4 in LSU, 2 in *RPB2*, 2 in *TEF1*, and 9 in *TUB2*. Relative to *C. kunmingensis*, differences were detected across all five loci, reaching 17 substitutions in *RPB2* and 16 in *TUB2*. Comparison with *C. chloroleuca* likewise revealed sequence differentiation at all loci examined, including 30 nucleotide substitutions in *TUB2*. The corresponding uncorrected p-distance values provide an additional quantitative measure of sequence divergence. These values are not interpreted as fixed numerical thresholds for species recognition; rather, their relevance lies in their agreement with the multilocus topology, individual gene genealogies, and diagnostic morphological differences.

Morphologically, *Clonostachys allendensis* differs from its closest phylogenetic relatives, *C. rhizophaga*, *C. kunmingensis*, and *C. chloroleuca*, through a distinctive combination of colony pigmentation and reproductive characters (Table 4). *Clonostachys allendensis* develops colonies that are initially white and subsequently become pale pink to salmon on PDA, together with yellowish to orange-yellow perithecia, cylindrical to lageniform or slightly ampulliform phialides measuring (8–)10–30(–35) × 2–4(–5) µm, and predominantly unicellular ellipsoidal to ovoid or subglobose conidia measuring (2.5–)3–8(–9) × (1.5–)2–3(–4) µm.

These characters distinguish the new species from *C. kunmingensis*, which develops pale yellow colonies with a pale orange reverse and possesses differentiated primary and secondary phialides and conidia [1]. *Clonostachys rhizophaga* also differs in the dimensions and organization of its conidiogenous structures, particularly in its generally longer primary phialides [4]. Comparison with *C. chloroleuca* is also taxonomically relevant because this species represents one of the phylogenetically related taxa identified in the multilocus analyses. *Clonostachys chloroleuca* exhibits differentiated primary and secondary conidiogenous structures and morphological characteristics that differ from the pale pink to salmon phenotype observed in *C. allendensis* [19]. The inclusion of *C. chloroleuca* in the comparative morphological assessment therefore complements its explicit consideration in the molecular analyses.

No single morphological character is considered independently diagnostic of *C. allendensis*. Instead, recognition of the new species is based on the particular combination of colony pigmentation, morphology and dimensions of the phialides and conidia, characteristics of the sexual morph, and its molecular differentiation from related taxa. This integrative interpretation is especially important within *Clonostachys*, where overlapping morphological character ranges may complicate species delimitation when phenotype is considered alone [2,4]. Consequently, the combined evidence from partitioned Maximum Likelihood inference, Bayesian analysis, partial genealogical concordance, sequence-level differentiation across multiple loci, and diagnostic morphology provides a stronger basis for recognizing *C. allendensis* than any single source of evidence considered independently.

Moderate phenotypic variation was observed among the isolates, particularly in colony pigmentation, perithecial size, and ascospore morphology. However, all isolates retained consistent micromorphological characteristics and clustered within the same phylogenetic lineage. Such variation is consistent with the phenotypic plasticity commonly reported in species of Hypocreales, where pigmentation, sporulation patterns, and reproductive structures may vary according to culture conditions, substrate composition, and environmental factors [4]. These observations support the interpretation that the isolates represent a single species exhibiting natural intraspecific variability rather than distinct taxa.

From an ecological perspective, the recovery of *C. allendensis* from pinto bean (*Phaseolus vulgaris* L.) straw is consistent with the broad ecological versatility reported for the genus. Species of *Clonostachys* occur in diverse substrates and ecological contexts and have been reported from soils, rhizospheres, plant tissues, and decomposing organic material, as well as in endophytic and mycoparasitic associations [1,5,6]. The three isolates representing the *C. allendensis* lineage were recovered from bean straw collected in the Los Llanos region of Durango, providing direct evidence of its association with this lignocellulosic agricultural residue under the environmental conditions of the study area. However, the present data do not demonstrate that *P. vulgaris* constitutes a specific host or preferred substrate for the species.

The discovery of *C. allendensis* on pinto bean straw expands current knowledge of the ecological distribution of *Clonostachys* species in Mexico. Previous studies in the country have focused mainly on isolates associated with soils, rhizospheres, endophytic communities, and biological control systems [21,22], whereas fungal communities colonizing agricultural residues in semi-arid environments remain comparatively underexplored. The occurrence of *Clonostachys* on lignocellulosic substrates such as bean straw suggests ecological adaptation to environments rich in structural plant polymers and supports the potential participation of these fungi in natural biomass decomposition processes.

This ecological association is further supported by previous characterization of native Clonostachys isolates recovered from bean straw in the same agricultural region. Cuevas-Jacquez et al. [22] reported cellulolytic activity and other extracellular enzymatic activities among these isolates, indicating their capacity to utilize structurally complex plant-derived substrates. Such characteristics are compatible with the colonization of lignocellulosic residues and may contribute to the establishment of these fungi on bean straw. Nevertheless, these observations should not be interpreted as evidence that *C. allendensis* is ecologically restricted to this substrate. Targeted sampling of soil, other agricultural residues, living plants, and surrounding natural vegetation will be necessary to determine its substrate range and ecological niche.

At present, the documented geographic occurrence of *C. allendensis* is limited to the type locality in the municipality of Guadalupe Victoria, Durango, Mexico. This should be regarded as its currently known distribution rather than as evidence of true geographic restriction or endemism. No systematic resampling specifically targeting *C. allendensis* has been conducted at the original collection site since the material analyzed in this study was obtained. Consequently, its current persistence at the type locality, local abundance, population structure, and broader geographic distribution remain unknown. Considering that other species of *Clonostachys* are known from diverse geographic regions and substrates [1,20], occurrence of *C. allendensis* at a single documented locality cannot by itself demonstrate ecological rarity or endemism.

Bean cultivation is recurrent in the Los Llanos region, and the continued production of bean straw results in repeated seasonal availability of substrates comparable to that from which *C. allendensis* was originally isolated [22]. The persistence of this substrate makes continued occurrence or recolonization of the fungus ecologically plausible. However, substrate continuity cannot be considered direct evidence that *C. allendensis* remains present at the original collection site. Confirmation will require systematic resampling of bean straw and surrounding soils across different cropping cycles and localities. Sampling of additional plant residues and natural substrates would further help determine whether the currently observed association reflects substrate preference or simply the ecological context in which the species was first detected.

The available information is likewise insufficient for a formal assessment of the conservation status of *C. allendensis*. Its presently restricted known distribution may reflect limited sampling rather than true rarity. Accordingly, the species should not currently be characterized as rare, endemic, threatened, or geographically restricted solely on the basis of the available collections. Future surveys across additional bean-producing areas of Durango and neighboring regions, together with repeated temporal sampling, will be necessary to determine its persistence and actual geographic distribution. Such studies would also allow evaluation of intraspecific genetic diversity among geographically separated populations and provide the information required for any future assessment of conservation status.

Species of *Clonostachys* have attracted increasing attention because of their ecological versatility and biotechnological potential, particularly in biological control, enzyme production, and lignocellulose degradation [5,7,23]. In this context, the comparatively higher extracellular enzymatic activity observed in the type strain, *C. allendensis* CM-CNRG_T (RC7) [22], suggests potential relevance in the transformation of agricultural residues. Although the present study focused primarily on taxonomic characterization, these findings provide a foundation for future studies exploring the enzymatic capabilities and possible biotechnological applications of this species in agricultural and environmental systems.

Nevertheless, functional characteristics documented for other members of Clonostachys should not automatically be extrapolated to *C. allendensis*. Although the isolates belonging to the lineage evaluated here exhibited enzymatic activities of biotechnological interest [22], further studies are required to characterize their ecological functions, substrate range, interactions with other microorganisms, persistence under field conditions, and potential applications. This distinction is important because the primary contribution of the present study is taxonomic, whereas ecological and biotechnological properties require independent experimental validation.

Overall, the congruence between molecular phylogeny, morphology, and ecological association supports the recognition of *Clonostachys allendensis* as a novel species within Bionectriaceae. The revised phylogenetic evidence strengthens this conclusion by demonstrating strong support under both Maximum Likelihood and Bayesian inference, predominant genealogical concordance across individual loci, and measurable sequence differentiation from the closest comparative taxa. At the same time, the ecological and geographic information currently available remains limited and should therefore be interpreted conservatively. The discovery of this species contributes to the growing knowledge of fungal diversity associated with agricultural residues in Mexico and emphasizes the importance of integrative taxonomic studies for documenting fungal biodiversity in underexplored semi-arid ecosystems.

## 5. Conclusions

The integrative analyses performed in this study support the recognition of *Clonostachys allendensis* sp. nov. as a distinct species within Bionectriaceae. Partitioned multilocus Maximum Likelihood and Bayesian analyses recovered RC2, RC5, and RC7 as a well-supported lineage distinct from closely related taxa, including *C. rhizophaga*, *C. chloroleuca*, and *C. kunmingensis*. Individual-locus analyses further provided partial genealogical concordance: ITS, LSU, *RPB2*, and *TEF1* recovered the three isolates as an exclusive lineage, whereas *TUB2* showed topological discordance but no strongly supported conflicting sister relationship involving RC7. Sequence-level differentiation across the five loci and diagnostic morphological characters provided additional complementary evidence for species delimitation.

The discovery of *C. allendensis* on pinto bean (*Phaseolus vulgaris* L.) straw expands current knowledge of fungal diversity associated with agricultural residues in semi-arid ecosystems of Mexico. However, its currently documented occurrence at a single geographic locality should not be interpreted as evidence of endemism or restricted distribution. Broader and repeated sampling will be necessary to determine its geographic range, ecological niche, substrate associations, and persistence in agricultural environments. Although isolates belonging to this lineage have exhibited extracellular enzymatic activities associated with the utilization of lignocellulosic substrates, the ecological functions and potential biotechnological applications of *C. allendensis* require further experimental evaluation.

Overall, this study demonstrates the value of integrating morphology, multilocus phylogenetic inference, genealogical evidence, and sequence-level differentiation for resolving species boundaries within *Clonostachys*. The recognition of *C. allendensis* contributes to documenting the still underexplored fungal diversity associated with agricultural residues in Mexico and provides a taxonomic foundation for future studies addressing its distribution, ecology, and functional potential.

## Figures and Tables

**Table 1:** Taxa, strain numbers, GenBank accession numbers, and corresponding references for the sequences included in the phylogenetic analyses.

Species	Strain	ITS	LSU	RPB2	TEF1	TUB2	Reference
*Bionectria levigata*	CBS 948.97	OQ910639	OQ910998	—	—	—	[4]
*Bionectria vesiculosa*	W2728b/HMAS 183151 ^T^	HM050304	HM050302	—	—	—	[4]
*Clonostachys agarwalii*	CBS 533.81 ^T^	OQ910526	OQ910885	OQ927604	OQ944540	OQ982571	[4]
*Clonostachys apocyni*	CBS 130.87	OQ910527	OQ910886	OQ927605	OQ944541	OQ982572	[4]
*Clonostachys aurantiaca*	CBS 124757 ^T^	OQ910531	OQ910890	—	OQ944545	—	[4]
*Clonostachys aureofulvella*	CBS 100980 ^T^	OQ910532	OQ910891	OQ927610	OQ944546	OQ982576	[4]
*Clonostachys australiana*	CBS 102421 ^T^	OQ910540	OQ910899	OQ927618	OQ944554	OQ982584	[4]
*Clonostachys bambusae*	CBS 139411 ^T^	OQ910542	OQ910901	OQ927620	OQ944556	OQ982586	[4]
*Clonostachys bambusae*	CBS 496.90	OQ910543	OQ910902	—	OQ944557	OQ982587	[4]
*Clonostachys buxicola*	CBS 102419 ^T^	OQ910544	OQ910903	OQ927622	OQ944558	OQ982588	[4]
*Clonostachys capitata*	CBS 218.93 ^T^	OQ910545	OQ910904	OQ927623	OQ944559	OQ982589	[4]
*Clonostachys chlorina*	CBS 287.90 ^T^	OQ910546	OQ910905	—	—	—	[4]
*Clonostachys chloroleuca*	CBS 100495	OQ910547	OQ910906	OQ927625	—	OQ982591	[4]
*Clonostachys chloroleuca*	CBS 141588 ^T^	OQ910549	OQ910908	—	OQ944563	—	[4]
*Clonostachys compactiuscula*	CBS 913.97 ^T^	OQ910567	OQ910926	OQ927644	OQ944580	OQ982606	[4]
*Clonostachys cylindrica*	CBS 101113 ^T^	OQ910569	OQ910928	—	OQ944582	—	[4]
*Clonostachys ellipsoidea*	CBS 175.76 ^T^	OQ910580	OQ910939	OQ927655	OQ944592	OQ982617	[4]
*Clonostachys eriocamporesii*	MFLUCC 19-0486 ^T^	MN699133	MN699128	—	—	—	[4]
*Clonostachys farinosa*	CBS 124067	OQ910593	OQ910952	—	—	—	[4]
*Clonostachys farinosa*	CBS 364.78 ^T^	OQ910614	OQ910973	OQ927685	OQ944626	OQ982649	[4]
*Clonostachys flava*	CBS 915.97 ^T^	OQ910619	OQ910978	OQ927690	OQ944631	OQ982654	[4]
*Clonostachys fujianensis*	CBS 127474 ^T^	OQ910620	OQ910979	OQ927691	OQ944632	OQ982655	[4]
*Clonostachys fusca*	CBS 207.93 ^T^	OQ910622	OQ910981	OQ927693	OQ944634	OQ982657	[4]
*Clonostachys garysamuelsii*	CBS 123964 ^T^	OQ910624	OQ910983	OQ927695	OQ944636	OQ982658	[4]
*Clonostachys grammicospora*	CBS 209.93 ^T^	OQ910625	OQ910984	—	—	—	[4]
*Clonostachys grammicosporopsis*	CBS 102834	OQ910626	OQ910985	OQ927697	OQ944638	OQ982660	[4]
*Clonostachys hongkongensis*	CBS 115291 ^T^	OQ910630	OQ910989	OQ927700	OQ944642	OQ982663	[4]
*Clonostachys hongkongensis*	CBS 116542	OQ910631	OQ910990	—	—	—	[4]
*Clonostachys kowhai*	CBS 461.95 ^T^	OQ910633	OQ910992	OQ927702	OQ944645	OQ982665	[4]
*Clonostachys krabiensis*	CBS 192.96	OQ910634	OQ910993	OQ927703	OQ944646	OQ982666	[4]
*Clonostachys kunmingensis*	CBS 101920	OQ910635	OQ910994	OQ927704	OQ944647	OQ982667	[4]
*Clonostachys longiphialidica*	CBS 112.87 ^T^	OQ910643	OQ911002	—	OQ944655	—	[4]
*Clonostachys lucifer*	CBS 100008 ^T^	OQ910644	OQ911003	OQ927713	OQ944656	OQ982675	[4]
*Clonostachys miodochialis*	CBS 997.69 ^T^	OQ910646	OQ911005	OQ927715	OQ944658	OQ982677	[4]
*Clonostachys moreaui*	CBS 127881	OQ910647	OQ911006	OQ927716	OQ944659	OQ982678	[4]
*Clonostachys oblongispora*	CBS 100285 ^T^	OQ910648	OQ911007	OQ927717	OQ944660	OQ982679	[4]
*Clonostachys obovatispora*	CBS 118752 ^T^	OQ910649	OQ911008	OQ927718	OQ944661	OQ982680	[4]
*Clonostachys palmae*	CBS 119.87 ^T^	OQ910650	OQ911009	OQ927719	OQ944662	OQ982681	[4]
*Clonostachys parasporodochialis*	CBS 192.93 ^T^	OQ910651	OQ911010	OQ927720	OQ944663	OQ982682	[4]
*Clonostachys penicillata*	CBS 148211	OQ910652	OQ911011	—	—	—	[4]
*Clonostachys penicillata*	CBS 729.87 ^T^	OQ910654	OQ911013	OQ927722	OQ944666	OQ982685	[4]
*Clonostachys pilosella*	CLLG19028 ^T^	—	MT248415	—	—	—	[4]
*Clonostachys pityrodes*	CBS 102033 ^T^	OQ910655	OQ911014	—	OQ944667	OQ982686	[4]
*Clonostachys pnagiana*	CLLG19041 ^T^	—	MT248416	—	—	—	[4]
*Clonostachys pseudochroleuca*	CBS 187.94 ^T^	OQ910665	OQ911024	OQ927733	OQ944677	OQ982696	[4]
*Clonostachys pseudostriata*	CBS 309.96	OQ910673	OQ911032	OQ927741	OQ944685	OQ982704	[4]
*Clonostachys ralfsii*	CBS 703.97 ^T^	OQ910684	OQ911043	OQ927752	OQ944696	AF358217	[4]
*Clonostachys reniformis*	CBS 695.86 ^T^	OQ910685	OQ911044	OQ927753	OQ944697	OQ982714	[4]
*Clonostachys rhizophaga*	CBS 202.37 ^T^	OQ910694	OQ911053	OQ927762	OQ944706	OQ982723	[4]
*Clonostachys rhizophaga*	CBS 229.48	OQ910695	OQ911054	OQ927763	—	OQ982724	[4]
*Clonostachys rogersoniana*	CBS 920.97 ^T^	OQ910711	OQ911070	OQ927779	OQ944723	OQ982740	[4]
*Clonostachys rosea*	CBS 100000	OQ910713	OQ911072	—	—	—	[4]
*Clonostachys rosea*	CBS 122171	OQ910718	OQ911077	OQ927786	—	OQ982747	[4]
*Clonostachys rosea*	CBS 710.86 ^T^	OQ910774	OQ911133	OQ927842	OQ944786	OQ982801	[4]
*Clonostachys rosea f. catenulata*	CBS 154.27 ^T^	OQ910800	OQ911159	—	—	—	[4]
*Clonostachys samuelsii*	CBS 699.97 ^T^	OQ910812	OQ911171	OQ927878	OQ944822	OQ982832	[4]
*Clonostachys solani*	CBS 697.88 ^T^	OQ910828	OQ911187	OQ927893	OQ944838	OQ982847	[4]
*Clonostachys solani f. nigrovirens*	CBS 183.30 ^T^	OQ910844	OQ911203	OQ927909	—	OQ982862	[4]
*Clonostachys swieteniae*	MFLUCC 18-0572 ^T^	MT215573	MT396164	—	—	—	[4]
*Clonostachys vacuolata*	CBS 191.93 ^T^	OQ910868	OQ911227	—	OQ944876	—	[4]
*Clonostachys venezuelae*	CBS 107.87 ^T^	OQ910869	OQ911228	OQ927932	—	OQ982884	[4]
*Clonostachys zelandiae-novae*	CBS 100977	—	—	OQ927933	—	—	[4]
*Clonostachys zelandiae-novae*	CBS 100979 ^T^	OQ910872	OQ911231	—	OQ944880	—	[4]
*Clonostachys allendensis*	RC2	**PQ596150**	**PX400599**	**PX436938**	**PX436932**	**PX436935**	This study
*Clonostachys allendensis*	RC5	**PQ596149**	**PX400598**	**PX436939**	**PX436933**	**PX436936**	This study
*Clonostachys allendensis*	RC7/CM-CNRG 1263 ^T^	**PQ596151**	**PX400600**	**PX436940**	**PX436934**	**PX436937**	This study
*Sesquicillium buxi*	CBS 202.69	—	—	—	—	OQ968098	[4]
*Sesquicillium buxi*	JW182006	OQ911264	OQ911325	OQ914820	OQ944500	OQ968104	[4]
*Sesquicillium intermediophialidicum*	CBS 685.96 ^T^	OQ911277	OQ911338	—	OQ944512	—	[4]
*Sesquicillium symmetricum*	CBS 124.79 ^T^	OQ911301	OQ911362	OQ914853	OQ944536	OQ968128	[4]

Note: GenBank accession numbers correspond to sequences included in the phylogenetic analysis. Bold accession numbers indicate sequences generated in this study. Superscript T indicates type or ex-type material. *Sesquicillium buxi*, *S. intermediophialidicum*, and *S. symmetricum* were used as outgroup taxa. Abbreviations: ITS, internal transcribed spacer; LSU, large subunit; RPB2, RNA polymerase II second-largest subunit; TEF1, translation elongation factor 1-alpha; TUB2, beta-tubulin.

**Table 2:** Genealogical concordance phylogenetic species recognition (GCPSR) assessment of *Clonostachys allendensis* RC7 based on individual Maximum Likelihood analyses of ITS, LSU, *RPB2*, *TEF1*, and *TUB2*.

Locus	RC7 Position	RC2–RC5–RC7	Bootstrap	Relationship with Closest Species	GCPSR Conflict
ITS	RC7 first groups with RC2, with RC5 joining subsequently.	Yes. RC2–RC5–RC7 form an exclusive clade.	66.2% for RC2–RC5–RC7; 24.8% for RC2 + RC7.	The clade is next associated with *C. kunmingensis*; *C. rhizophaga* CBS 229.48 occurs at a subsequent node with very low support.	No strongly supported conflict. Compatible with GCPSR, although support is moderate.
LSU	RC7 first groups with RC2, with RC5 joining subsequently.	Yes. RC2–RC5–RC7 form an exclusive clade.	6.4% for RC2–RC5–RC7; 12.6% for RC2 + RC7.	The clade occurs close to *C. rhizophaga* CBS 229.48 and CBS 202.37.	Unresolved. The topology is compatible with GCPSR, but support is too low to provide strong evidence of genealogical concordance.
*RPB2*	RC7 first groups with RC5, with RC2 joining subsequently.	Yes. RC2–RC5–RC7 form an exclusive clade.	48.4% for RC2–RC5–RC7; 23.6% for RC5 + RC7.	The group is first associated with *C. chloroleuca* CBS 141588 and subsequently with *C. rhizophaga* CBS 202.37.	No strongly supported conflict. Compatible with GCPSR, but with low support.
*TEF1*	RC7 first groups with RC2, with RC5 joining subsequently.	Yes. RC2–RC5–RC7 form an exclusive clade.	86.8% for RC2–RC5–RC7; 31.8% for RC2 + RC7.	The RC2–RC5–RC7 clade is sister to the *C. rhizophaga* CBS 202.37 + CBS 229.48 clade; the combined node has 90.6% support.	Strong support and no conflict. This locus provided the clearest signal of genealogical concordance.
*TUB2*	RC7 does not form an exclusive clade with RC2 and RC5; RC2 + RC5 group together, whereas RC7 is topologically associated with *C. rhizophaga* CBS 202.37.	No. The clade containing RC2, RC5, and RC7 also includes *C. rhizophaga* CBS 229.48 and CBS 202.37.	94.75% for the broader RC2 + RC5 + RC7 + *C. rhizophaga* clade; 4.75% for *C. rhizophaga* CBS 202.37 + RC7; 22.0% for RC2 + RC5.	*C. rhizophaga* is intercalated within the group containing the three isolates.	Topological discordance is present. However, the immediate RC7 + *C. rhizophaga* CBS 202.37 association has very low support (4.75%), and therefore does not constitute a strongly supported conflicting relationship.

**Table 3:** Pairwise nucleotide differences and uncorrected p-distances between *Clonostachys* allendensis RC7 and selected phylogenetically related species across the five loci analyzed.

Locus	Species Compared with RC7	Strain	Nucleotide Differences (n)	Uncorrected p-Distance
ITS	*Clonostachys rhizophaga*	CBS 202.37	4	0.0080
ITS	*Clonostachys rhizophaga*	CBS 229.48	2	0.0040
ITS	*Clonostachys kunmingensis*	CBS 101920	4	0.0080
ITS	*Clonostachys chloroleuca*	CBS 100495	4	0.0080
LSU	*Clonostachys rhizophaga*	CBS 202.37	4	0.0050
LSU	*Clonostachys rhizophaga*	CBS 229.48	4	0.0050
LSU	*Clonostachys kunmingensis*	CBS 101920	5	0.0063
LSU	*Clonostachys chloroleuca*	CBS 141588	7	0.0088
LSU	*Clonostachys chloroleuca*	CBS 100495	7	0.0088
RPB2	*Clonostachys rhizophaga*	CBS 202.37	2	0.0027
RPB2	*Clonostachys kunmingensis*	CBS 101920	17	0.0226
RPB2	*Clonostachys chloroleuca*	CBS 141588	6	0.0080
TEF1	*Clonostachys rhizophaga*	CBS 202.37	2	0.0025
TEF1	*Clonostachys rhizophaga*	CBS 229.48	2	0.0025
TEF1	*Clonostachys kunmingensis*	CBS 101920	5	0.0062
TEF1	*Clonostachys chloroleuca*	CBS 100495	8	0.0099
TUB2	*Clonostachys rhizophaga*	CBS 202.37	9	0.0085
TUB2	*Clonostachys rhizophaga*	CBS 229.48	9	0.0085
TUB2	*Clonostachys kunmingensis*	CBS 101920	16	0.0152
TUB2	*Clonostachys chloroleuca*	CBS 100495	30	0.0286

Note: RC7 is the ex-type strain of *Clonostachys allendensis* (CM-CNRG 1263). Uncorrected p-distance represents the proportion of nucleotide differences per compared site. Comparisons are reported only for strains/loci included in the corresponding single-locus datasets.

**Table 4:** Morphological comparison of *Clonostachys allendensis* and its closest phylogenetic relatives.

Character	*C. allendensis* (This Study)	*C. rhizophaga* [4]	*C. kunmingensis* [1]	*C. chloroleuca* [19]
Colony pigmentation on PDA	Initially white, becoming pale pink to salmon at maturity	White to pale yellow	Pale yellow; reverse pale orange	Greenish-white; reverse pale yellow
Perithecia	Present; yellowish to orange-yellow, (120–)150–250(–300) µm diam.	Present	Not observed	Present
Primary phialides	Cylindrical to lageniform or slightly ampulliform, (8–)10–30(–35) × 2–4(–5) µm	Divergent, 15.6–48.2 × 2.2–3.2 µm	Cylindrical, slightly tapering toward the tip, (14.2–)19.1–36.4(–52.6) × (2–)2.5–3.5(–3.9) µm	Generally, in whorls of 2–5, 12.7–36.5 × 1.85–2.8 µm
Secondary phialides	Not differentiated into a distinct secondary morph in the present description	Adpressed or divergent, 5.8–25.2 × 2.2–3.2 µm	Divergent or adpressed, (5.6–)8.0–17.5(–25) × (2–)2.5–3.2(–4) µm	Adpressed, in whorls of up to 5, 9.9–18.5 × 1.3–3.5 µm
Conidia	Predominantly unicellular, ellipsoidal to ovoid or subglobose, (2.5–)3–8(–9) × (1.5–)2–3(–4) µm	Aseptate, ellipsoidal to cylindrical, 4.8–9 × 2.4–4.2 µm	Primary: oblong to cylindrical, (6–)6.7–11.2(–14) × (2–)2.3–4.5(–4.8) µm; secondary: slightly curved, (4–)4.2–8.5(–9) × (2.2–)2.5–4(–4.5) µm	Hyaline, ellipsoidal, slightly curved or asymmetrical; 5.9–9.2 × 4.9–6.6 µm
Sexual morph	Present	Present	Undetermined	Present
Phylogenetic position	Independent lineage supported by ITS, LSU, *TEF1*, *TUB2*, and *RPB2*	Closely related species	Closely related species	Closely related species

## Data Availability

The sequence data generated in this study are publicly available in the GenBank database under the accession numbers PQ596149- PQ596151, PX436932-PX436940, and PX400598- PX400600. Additional data supporting the findings of this study are available within the article.

## Supplementary Materials

The following supporting information can be downloaded at: https://www.mdpi.com/article/10.3390/microorganisms14091962/s1, Figure S1: ITS region maximum likelihood phylogeny inferred from the corresponding single-locus alignment using the K2P+G substitution model. Nodal labels indicate bootstrap support values with 500 replicates; branch lengths are proportional to nucleotide substitutions per site as indicated by the scale bar. The topology was evaluated under the GCPSR framework described in the revised manuscript. Figure S2: LSU maximum Likelihood phylogeny inferred from the corresponding single-locus alignment using the K2P+G+I substitution model. Nodal labels indicate bootstrap support values with 500 replicates; branch lengths are proportional to nucleotide substitutions per site as indicated by the scale bar. The topology was evaluated under the GCPSR framework described in the revised manuscript. Figure S3: *RPB2* Maximum Likelihood phylogeny inferred from the corresponding single-locus alignment using the T92+G+I substitution model. Nodal labels indicate bootstrap support values with 500 replicates; branch lengths are proportional to nucleotide substitutions per site as indicated by the scale bar. The topology was evaluated under the GCPSR framework described in the revised manuscript. Figure S4: *TEF1* maximum likelihood phylogeny inferred from the corresponding single-locus alignment using the GTR+G+I substitution model. Nodal labels indicate bootstrap support values with 500 replicates; branch lengths are proportional to nucleotide substitutions per site as indicated by the scale bar. The topology was evaluated under the GCPSR framework described in the revised manuscript. Figure S5: *TUB2* maximum likelihood phylogeny inferred from the corresponding single-locus alignment using the K2P+G substitution model. Nodal labels indicate bootstrap support values with 500 replicates; branch lengths are proportional to nucleotide substitutions per site as indicated by the scale bar. The topology was evaluated under the GCPSR framework described in the revised manuscript.
