# Supplementary material for: Clonostachys allendensis sp. nov.: A New Species Isolated from Bean Straw in Durango, Mexico
Source: Microorganisms. 2026 Sep 5;14(9):1962. doi: 10.3390/microorganisms14091962 (PMC13609091; doi:10.3390/microorganisms14091962)
Supplement: Supplementary file 1 [file microorganisms-14-01962-s001.zip › microorganisms-4498789-supplementary.pdf]

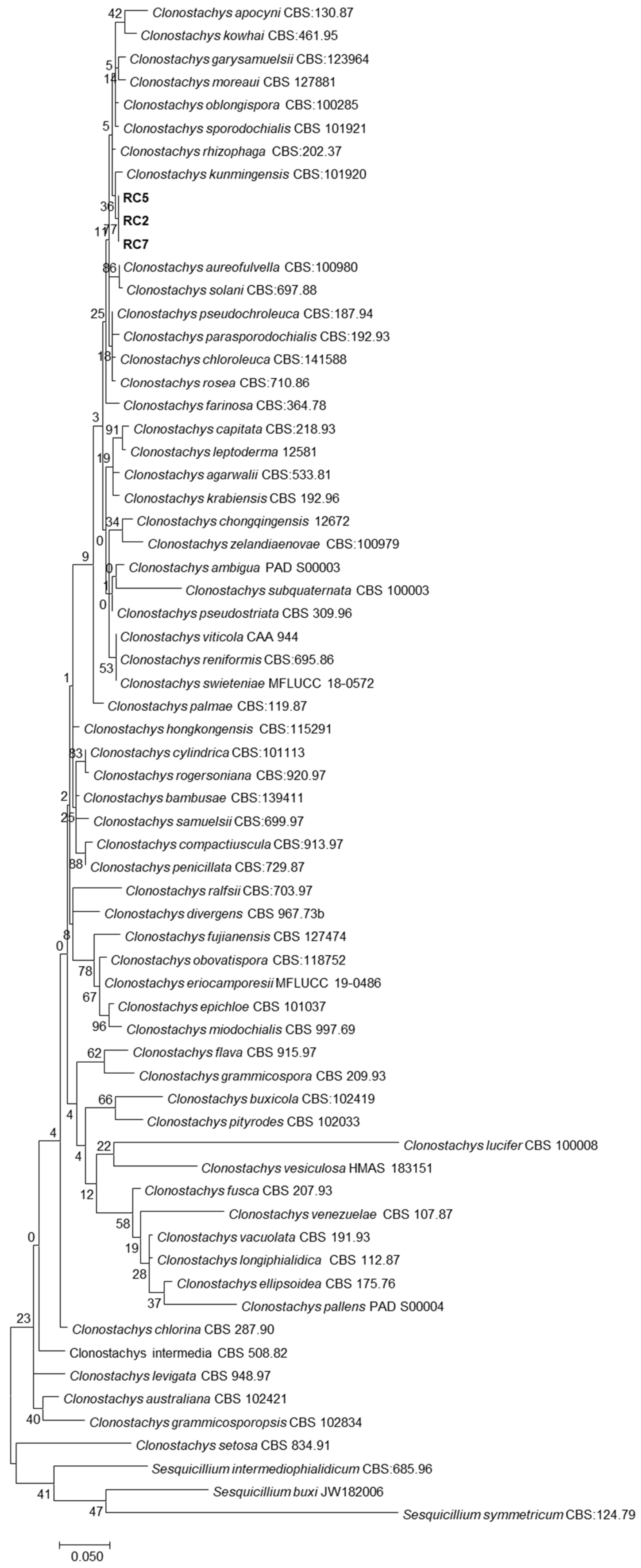

Figure S1. ITS region maximum likelihood phylogeny inferred from the corresponding single-locus alignment using the K2P+G substitution model. Nodal labels indicate bootstrap support values with 500 replicates; branch lengths are proportional to nucleotide substitutions per site as indicated by the scale bar. The topology was evaluated under the GCPSR framework described in the revised manuscript.

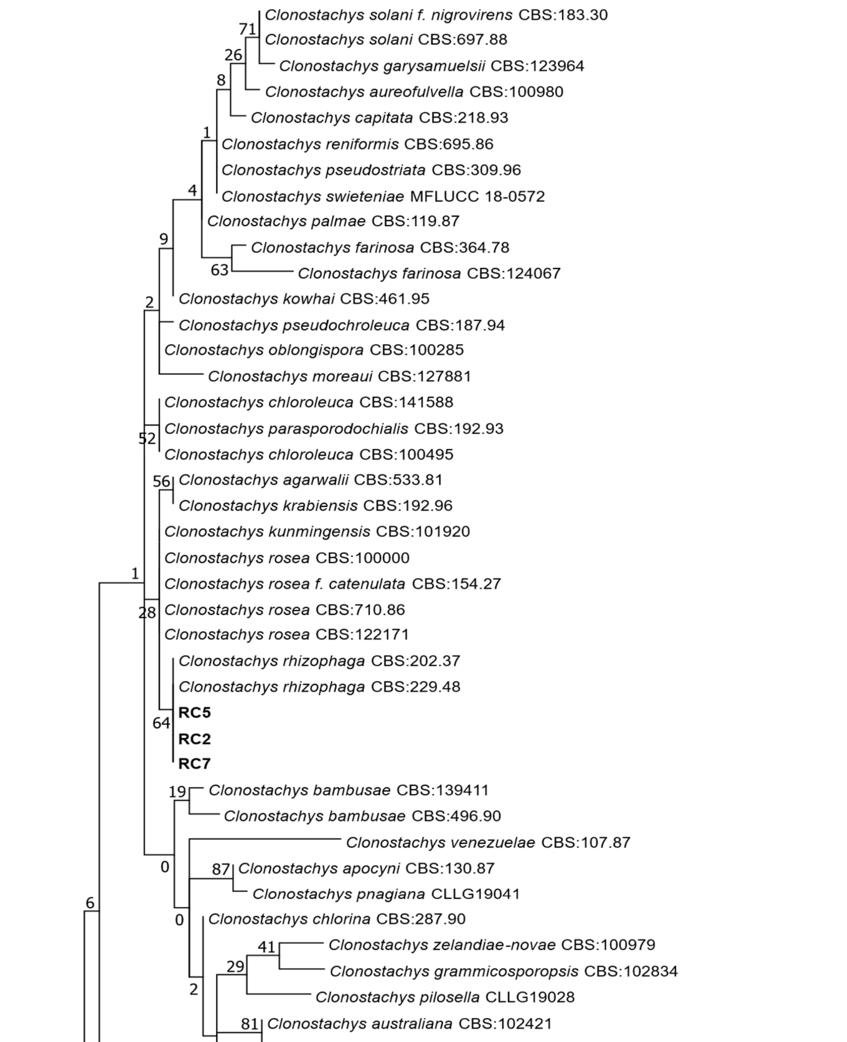

Figure S2. LSU maximum Likelihood phylogeny inferred from the corresponding single-locus alignment using the K2P+G+I substitution model. Nodal labels indicate bootstrap support values with 500 replicates; branch lengths are proportional to nucleotide substitutions per site as indicated by the scale bar. The topology was evaluated under the GCPSR framework described in the revised manuscript.

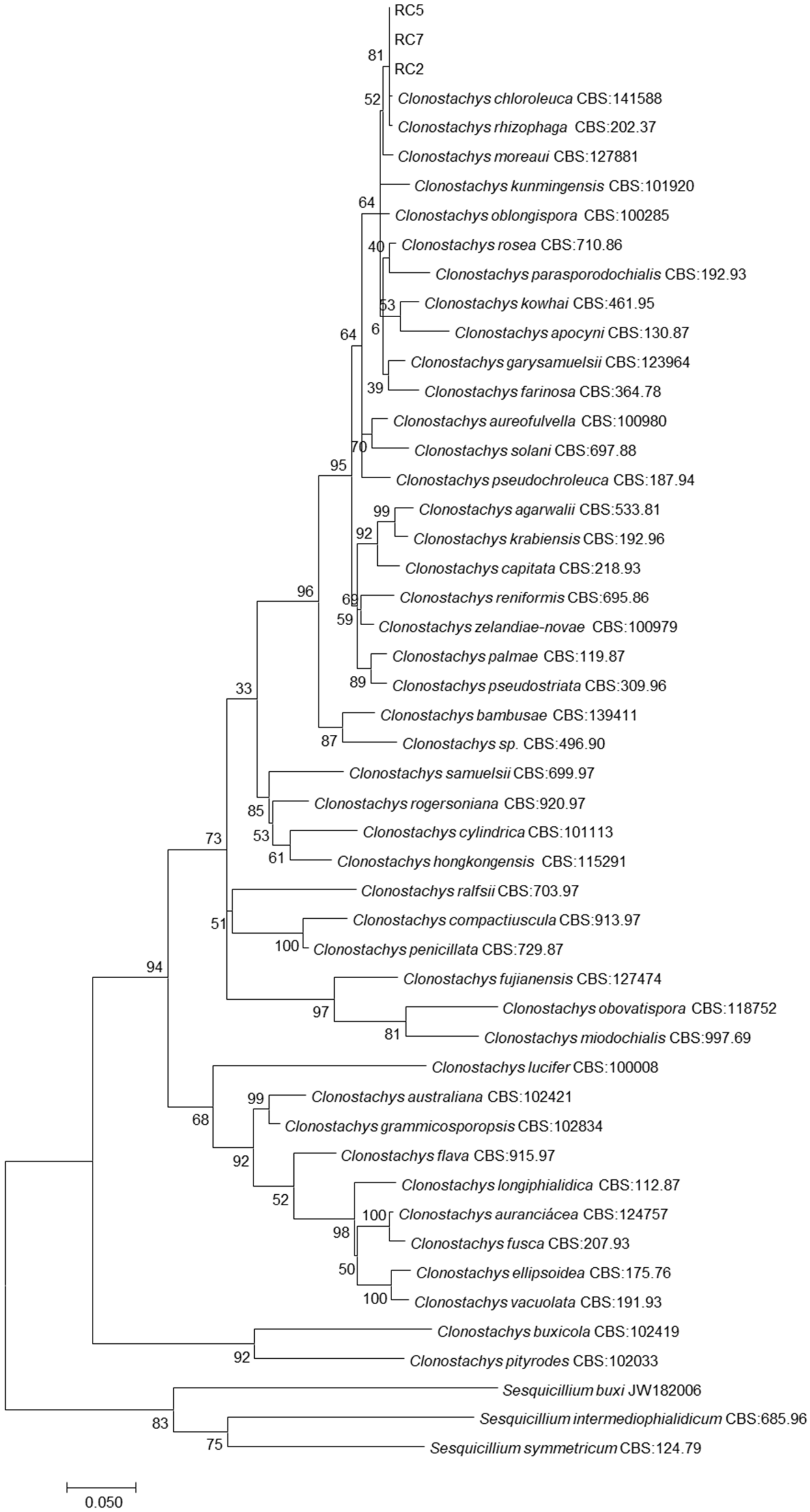

Figure S3. *RPB2* Maximum Likelihood phylogeny inferred from the corresponding single-locus alignment using the T92+G+I substitution model. Nodal labels indicate bootstrap support values with 500 replicates; branch lengths are proportional to nucleotide substitutions per site as indicated by the scale bar. The topology was evaluated under the GCPSR framework described in the revised manuscript.

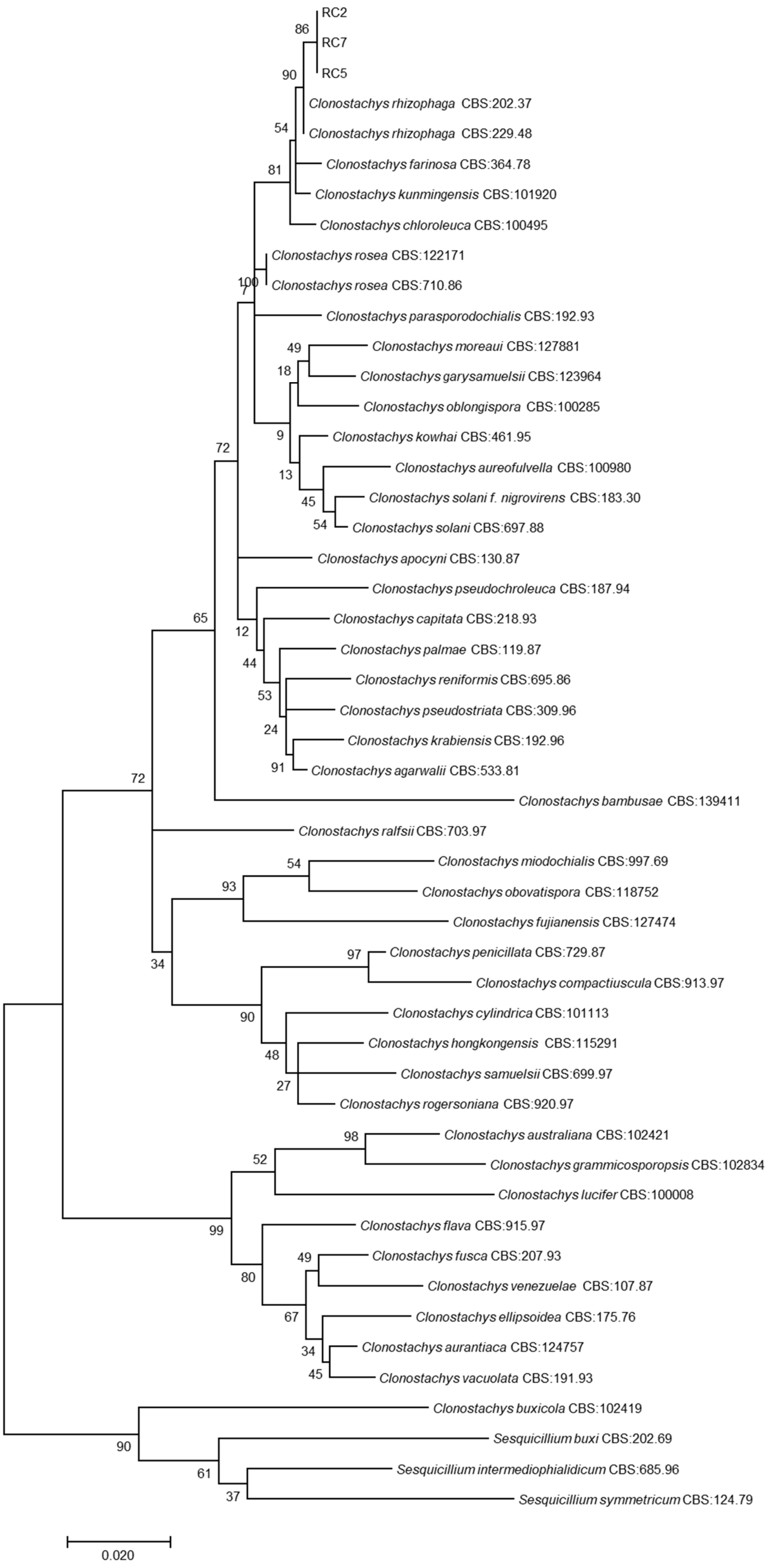

Figure S4. *TEF1* maximum likelihood phylogeny inferred from the corresponding single-locus alignment using the GTR+G+I substitution model. Nodal labels indicate bootstrap support values with 500 replicates; branch lengths are proportional to nucleotide substitutions per site as indicated by the scale bar. The topology was evaluated under the GCPSR framework described in the revised manuscript.

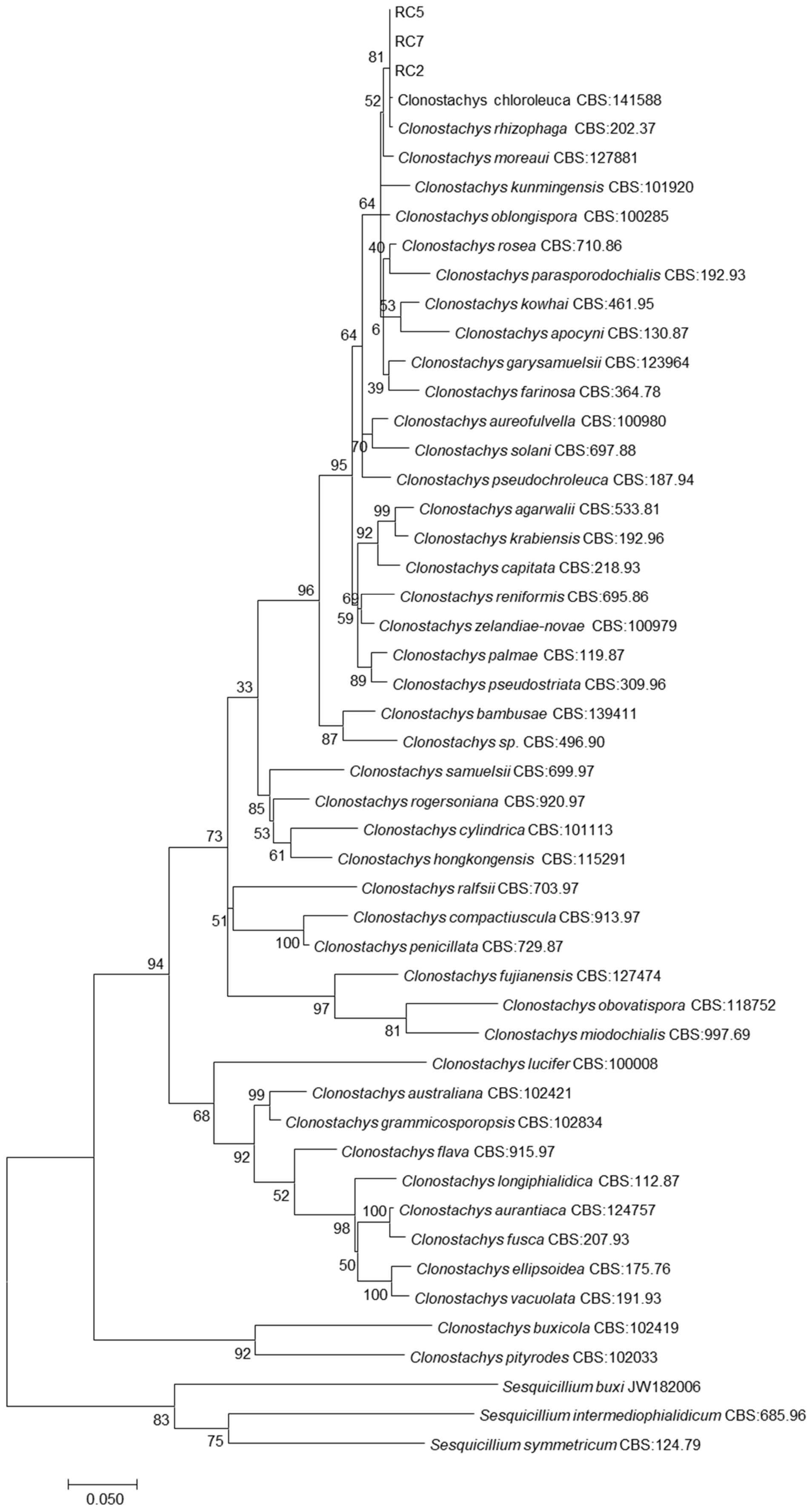

Figure S5. *TUB2* maximum likelihood phylogeny inferred from the corresponding single-locus alignment using the K2P+G substitution model. Nodal labels indicate bootstrap support values with 500 replicates; branch lengths are proportional to nucleotide substitutions per site as indicated by the scale bar. The topology was evaluated under the GCPSR framework described in the revised manuscript.
